# The efficacy of whole human genome capture on ancient dental calculus and dentin

**DOI:** 10.1002/ajpa.23763

**Published:** 2018-12-26

**Authors:** Kirsten A. Ziesemer, Jazmín Ramos‐Madrigal, Allison E. Mann, Bernd W. Brandt, Krithivasan Sankaranarayanan, Andrew T. Ozga, Menno Hoogland, Courtney A. Hofman, Domingo C. Salazar‐García, Bruno Frohlich, George R. Milner, Anne C. Stone, Mark Aldenderfer, Cecil M. Lewis, Corinne L. Hofman, Christina Warinner, Hannes Schroeder

**Affiliations:** ^1^ Faculty of Archaeology Leiden University Leiden The Netherlands; ^2^ Natural History Museum of Denmark University of Copenhagen Copenhagen Denmark; ^3^ Laboratories of Molecular Anthropology and Microbiome Research and Department of Anthropology University of Oklahoma Norman Oklahoma; ^4^ Department of Preventive Dentistry Academic Centre for Dentistry Amsterdam, University of Amsterdam and VU University Amsterdam Amsterdam The Netherlands; ^5^ Department of Microbiology and Plant Biology University of Oklahoma Norman Oklahoma; ^6^ School of Human Evolution and Social Change Arizona State University Tempe Arizona; ^7^ Institute for Human Origins Arizona State University Tempe Arizona; ^8^ Center for Evolution and Medicine Arizona State University Tempe Arizona; ^9^ Department of Archaeogenetics Max Planck Institute for the Science of Human History Jena Germany; ^10^ Grupo de Investigación en Prehistoria IT‐622‐13 (UPV‐EHU)/IKERBASQUE‐Basque Foundation for Science Vitoria Spain; ^11^ Department of Anthropology Hanover New Hampshire; ^12^ Department of Anthropology Pennsylvania State University University Park Pennsylvania; ^13^ Department of Anthropology and Heritage Studies University of California Merced California

**Keywords:** ancient DNA, genomics, hybridization capture, target enrichment

## Abstract

**Objectives:**

Dental calculus is among the richest known sources of ancient DNA in the archaeological record. Although most DNA within calculus is microbial, it has been shown to contain sufficient human DNA for the targeted retrieval of whole mitochondrial genomes. Here, we explore whether calculus is also a viable substrate for whole human genome recovery using targeted enrichment techniques.

**Materials and methods:**

Total DNA extracted from 24 paired archaeological human dentin and calculus samples was subjected to whole human genome enrichment using in‐solution hybridization capture and high‐throughput sequencing.

**Results:**

Total DNA from calculus exceeded that of dentin in all cases, and although the proportion of human DNA was generally lower in calculus, the absolute human DNA content of calculus and dentin was not significantly different. Whole genome enrichment resulted in up to four‐fold enrichment of the human endogenous DNA content for both dentin and dental calculus libraries, albeit with some loss in complexity. Recovering more on‐target reads for the same sequencing effort generally improved the quality of downstream analyses, such as sex and ancestry estimation. For nonhuman DNA, comparison of phylum‐level microbial community structure revealed few differences between precapture and postcapture libraries, indicating that off‐target sequences in human genome‐enriched calculus libraries may still be useful for oral microbiome reconstruction.

**Discussion:**

While ancient human dental calculus does contain endogenous human DNA sequences, their relative proportion is low when compared with other skeletal tissues. Whole genome enrichment can help increase the proportion of recovered human reads, but in this instance enrichment efficiency was relatively low when compared with other forms of capture. We conclude that further optimization is necessary before the method can be routinely applied to archaeological samples.

## 1 INTRODUCTION

The development and application of sequence capture technology has greatly increased the number of archaeological samples that are accessible for genomic studies (e.g., Carpenter et al., 2013; Fu, Mittnik, et al., 2013; Haak et al., 2015; Schroeder et al., 2015). Typically, the majority of DNA in a given archaeological sample is exogenous (i.e., postmortem environmental) in origin, making untargeted sequencing of these samples inefficient and expensive, with the exception of extraordinarily well‐preserved samples and bone elements, such as the petrous bone (Gamba et al., 2014). Targeted sequence capture allows for the selective enrichment of endogenous ancient DNA (aDNA) sequences prior to sequencing, thereby increasing the proportion of desired, on‐target molecules in the sequencing run. Sequence capture additionally reduces the amount of material required for destructive analyses, and decreases the experimental workload and cost of aDNA analysis (Ávila‐Arcos et al., 2011; Carpenter et al., 2013). To date, targeted enrichment of archaeological specimens has resulted in the successful retrieval of ancient mitochondrial genomes (e.g., Briggs et al., 2009; Llamas et al., 2016; Ozga et al., 2016; Slon et al., 2016), ancient pathogen genomes (Bos et al., 2014; Spyrou et al., 2016; Vågene et al., 2018), human genome‐wide SNPs (Haak et al., 2015), partial or whole exomes (Burbano et al., 2010; Da Fonseca et al., 2015), entire chromosomes (Cruz‐Dávalos et al., 2017; Fu, Meyer, et al., 2013), and partial nuclear genomes (Carpenter et al., 2013; Schroeder et al., 2015).

The majority of these aDNA capture studies have focused on either archaeological bone or dentin as sample material. However, host DNA preservation in these tissues is highly variable (Damgaard et al., 2015; Gamba et al., 2014), and destructive analysis of skeletal remains may be restricted or not permitted in some cases, making archaeogenetic analysis of these populations challenging. Recent research on ancient dental calculus (calcified dental plaque) has shown that it is the richest known source of ancient DNA in the archaeological record, exceeding the DNA content found in bone and dentin by more than an order of magnitude (Mann et al., 2018; Ozga et al., 2016; Warinner, Rodrigues, et al., 2014). Consequently, dental calculus is potentially valuable for studies of ancient and degraded samples, where DNA preservation is limited. Moreover, as dental calculus is a calcified bacterial biofilm, not a human tissue, it might be subject to fewer restrictions with respect to destructive sampling (Ozga et al., 2016).

The vast majority of DNA in ancient dental calculus is microbial in origin, which explains why the majority of ancient DNA research on dental calculus has focused primarily on this component (Adler et al., 2013; Warinner, Rodrigues, et al., 2014; Warinner, Speller, & Collins, 2014; Warinner, Speller, Collins, & Lewis Jr, 2015; Weyrich et al., 2017; Ziesemer et al., 2015). However, a small but consistent proportion (around 0.1%) of DNA in ancient dental calculus comes from the host (Mann et al., 2018; Ozga et al., 2016; Warinner, Rodrigues, et al., 2014). The mechanisms of human DNA incorporation into dental calculus are not well understood, but the primary source of host DNA is likely saliva and gingival crevicular fluid (Jin & Yip, 2002). Potential cell types contributing host DNA include white blood cells (e.g., neutrophils, basophils, eosinophils, monocytes, and lymphocytes) and oral epithelial cells (Mann et al., 2018; Warinner, Rodrigues, et al., 2014). Previous proteomic analysis of ancient and modern dental calculus identified a high proportion of immune proteins, particularly from neutrophils, suggesting that human DNA may enter dental calculus as a result of inflammation‐related immunological activity, including the release of neutrophil extracellular traps (NETosis) (Warinner, Rodrigues, et al., 2014).

Archaeological dental calculus has been shown to contain sufficient mitochondrial DNA for full mitogenome reconstruction (Ozga et al., 2016); however, mitogenomes only provide maternal ancestry information. In contrast, genome‐wide sequence data provide significantly more information that can be used to determine the sex of individuals (Skoglund et al., 2013), infer genome‐wide ancestry and admixture patterns (e.g., Skoglund et al., 2014), establish kinship and genetic relationships (Sikora et al., 2017), and provide evidence for natural selection and human environment interactions (e.g., Jeong et al., 2016). Establishing whether ancient dental calculus can serve as a viable source of genome‐wide nuclear human DNA is thus important in order to evaluate its potential for future population genetics studies.

**Figure 1:**
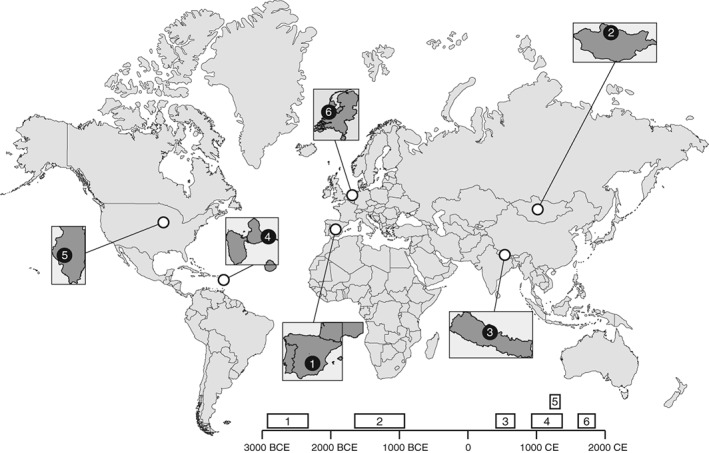
Location of archaeological sites. We selected 24 well‐preserved paired dentin and dental calculus samples from six archaeological sites spanning three continents. The sites include (1) Camino del Molino, Spain (C53 and C214); (2) Khövsgöl, Arbulag Soum, Mongolia (H10 and H24); (3) Samdzong, Nepal (S40 and S41); (4) Anse à la gourde, Guadeloupe (F349A and F1948), (5) Norris Farms, Illinois, USA (NF47 and NF217); and (6) Middenbeemster, the Netherlands (S108 and S454)

In this article, we perform whole genome enrichment (WGE) on 24 paired archaeological human dentin and dental calculus samples (Figure 1) that had been previously shown to be well preserved (Mann et al., 2018). In total, we generated approximately 600 million paired‐end reads and characterized the quantity and quality of the human genetic data obtained. The samples were sourced from diverse contexts to assess if patterns of preservation in dental calculus vary across time and space, and to evaluate the performance of WGE on samples with varying levels of preservation. The enrichment was performed using the MYbaits WGE kit (Arbor Biosciences, MI), which uses biotinylated RNA “bait” to capture the human DNA molecules in ancient DNA libraries (Enk et al., 2014).

We find that although dental calculus is an excellent source of both microbial and human endogenous DNA, the relative proportion of human DNA is consistently quite low, making efficient WGE challenging. In this study, we observed only modest enrichments of up to four‐fold, which is relatively low when compared with previously published enrichment rates for mitochondrial genome capture (Ozga et al., 2016) or selected SNPs (e.g., Mathieson et al., 2015). Additionally, we find that capture enrichment of dental calculus results in the biased recovery of human reads with significantly higher GC content. Nevertheless, in the absence of other available skeletal tissues, or when the endogenous content in other tissues is low, dental calculus can serve as a viable source of nuclear human DNA. Surprisingly, although the total number of microbial 16S rRNA gene reads did not decrease after capture, the proportion of these reads clustering into operational taxonomic units (OTUs) did. Comparison of the microbial community structure in dental calculus samples at the phylum‐level, however, revealed few differences between precapture and postcapture libraries, indicating that off‐target sequences in human genome‐enriched dental calculus libraries may still be useful for ancient oral microbiome reconstruction. Overall, we find that dental calculus is a valuable source of human DNA; however, to unlock the full potential of dental calculus for genome‐wide analyses, current DNA enrichment techniques require further optimization.

## 2 MATERIALS AND METHODS

### 2.1 Samples

We analyzed 24 paired human dental calculus and dentin samples from six geographically and temporally diverse sites, with two individuals analyzed per site (Figure 1; Supporting Information Table S1). The samples and sites were selected to reflect broad geographic distribution and temporal coverage: (1) Camino del Molino, Spain (C53 and C214, ca., 2,340–2,920 BCE; Ziesemer et al., 2015), (2) Arbulag Soum, Khövsgöl, Mongolia (H10 and H24, ca., 2,000 BCE; Littleton et al., 2012), (3) Samdzong, Nepal (S40 and S41, ca., 400–650 CE; Ziesemer et al., 2015; Jeong et al., 2016), (4) Anse à la Gourde, Guadeloupe (F349A and F1948, ca., 975–1,395 CE; Ziesemer et al., 2015), (5) Norris Farms, Illinois, USA (NF47 and NF217, ca., 1,300 CE; Ozga et al., 2016), (6) Middenbeemster, the Netherlands (S108 and S454, 1,611–1866 CE; Ziesemer et al., 2015). The same sample set was also evaluated in a separate study on the differential preservation of ancient DNA in dental calculus and dentine (Mann et al., 2018).

### 2.2 DNA extraction

All samples were extracted in dedicated ancient DNA facilities at the Laboratories of Molecular Anthropology and Microbiome Research (LMAMR) in Norman, Oklahoma. The LMAMR lab operates in accordance with established contamination control precautions and workflows, as previously described (Ziesemer et al., 2015). Prior to extraction, the surface of the tooth was washed with a 2% NaOCl solution, followed by molecular biology grade water. Dental calculus was then removed from the tooth using a dental scaler, and the tooth root was separated from the crown using a Dremel rotary tool. The tooth root and calculus were further decontaminated by UV irradiation in a Crosslinker for 1 min on each side. DNA extraction was performed as described by Ziesemer et al. (2015). In brief, 10–20 mg of dental calculus and approximately 100 mg of dentin were crushed to a coarse powder and washed in 1 ml 0.5 M EDTA under rotation for 15 minutes to remove loosely‐bound contaminants. Following centrifugation, the supernatant was removed, and the decontaminated pellet was digested in a solution of 0.45 M EDTA and 10% proteinase K with heating at 37–55°C for 8–12 hr, followed by room temperature incubation for 5 days until digestion was complete. For dentin samples, the digestion buffer was refreshed after 2 days to avoid saturation of EDTA chelation, and the two buffer fractions were combined after digestion completion. For all samples, cell pellet debris was separated by centrifugation and the DNA‐containing supernatant was further extracted using a phenol/chloroform approach (Warinner, et al., 2014), followed by DNA purification and concentration using a modified Qiagen MinElute silica spin column protocol (Dabney et al., 2013). Extracted DNA was eluted twice into 30 μl of EB for a combined volume of 60 μl, and immediately quantified after extraction using a Qubit fluorometer 2.0 High Sensitivity assay (Life Technologies). Subsequent DNA quantification performed later in a separate study measured higher concentrations, likely due to evaporation (Mann et al., 2018).

### 2.3 Illumina library preparation

Approximately 100 ng of total DNA was built into each Illumina shotgun library at the LMAMR lab using the NEBNext DNA Library Prep Master Set (E6070; New England Biolabs) following manufacturer's instructions, with minor modifications. In brief, end‐repair was performed in 50 μl reactions with up to 30 μl of DNA extract for each sample. The end‐repair cocktail was incubated for 20 min at 12°C and 15 min at 37°C, purified using Qiagen MinElute silica spin columns, and eluted in 30 μl. After end‐repair, blunt‐end Illumina‐specific adapters (Meyer & Kircher, 2010) were ligated to end‐repaired DNA in 50 μl reactions. The reaction was incubated for 15 min at 20°C and purified using Qiagen QiaQuick columns before elution in 30 μl EB. The adapter fill‐in reaction was performed in a final volume of 50 μl and incubated for 20 min at 37°C followed by 20 min at 80°C. Libraries were amplified and indexed at the Center for Geogenetics in Copenhagen, Denmark, using a dual‐indexing approach (Kircher, Sawyer, & Meyer, 2012) in a 50 μl PCR reaction, using 15 μl of library template, 25 μl of a 2× KAPA HiFi Uracil+ Master Mix, 5.5 μl H_2_O, 1.5 μl DMSO, 1 μl BSA (20 mg/ml), and 1 μl each of a forward and reverse indexing primer (10 μM). Thermocycling conditions were as follows: 5 min at 98°C, followed by 10–12 cycles of 15 sec at 98°C, 20 sec at 60°C, 20 sec at 72°C, and a final 1 min elongation step at 72°C. Amplified libraries were purified using Agencourt AMPure XP beads, and eluted in 30 μl EB. The concentration/molarity and size distribution of the libraries was estimated using an Agilent Bioanalyzer.

### 2.4 MYBait human whole genome enrichment

Whole genome capture was performed at the Center for Geogenetics using the MYbaits Whole Genome Enrichment kit, following manufacturer's instructions (MYbaits protocol version 2.3.1 May 2014). In brief, 6 μl of the adapter‐ligated genomic DNA library, 2.5 μl of 1 μg/μl Human Cot‐1 DNA, 2.5 μl of 1 μg/μl Salmon Sperm DNA and 0.6 μl of proprietary Blocking Agent were combined and incubated for 5 min at 95°C to denature the DNA. The hybridization master mix consisting of 20 μl of 20× SSPE, 0.8 μl 500 mM EDTA, 8 μl of 50× Denhardt's Solution and 0.8 μl of 10% SDS was preheated for 3 min at 65°C, and 5 μl of SUPERase‐In (20 U/μl) and 1 μl of biotinylated RNA baits were also preheated for 2 min at 65°C. While keeping the tubes at 65°C, the denatured DNA mix was added to the preheated biotinylated RNA baits mixture. Then, the hybridization mix was added to the biotinylated RNA baits/denatured DNA mixture. This mixture was hybridized at 65°C for 16 hr. The hybridized RNA baits were then isolated from the unbound genomic DNA using Dynabeads MyOne Streptavidin C1 beads. A higher wash stringency, using a five‐fold dilution of 340 μl 0.1× SSC and 1,360 μl 0.1% SDS, was used during the recovery of the captured targets. After three washes, the beads were resuspended in 20 μl of a buffer solution of 1 M NaCl, 10 mM Tris–HCl (pH 7.5), and 1 mM EDTA, and incubated at 65°C for 2 min. This entire volume was transferred to the hybridization solution at 65°C for 45 min, after which it was immediately pelleted with a magnetic particle stand for 2 min. Preheated 0.1× SSC and 0.1% SDS (500 μl) was added to the mixture and incubated for 5 min at 65°C. The beads were pelleted with a magnetic particle stand for 2 min to remove the supernatant. This step was repeated twice for a total of three washes. The enriched DNA was eluted in 30 μl of molecular biology grade water. The postcapture amplification was similar to the indexing PCR with 15 μl of library template, 25 μl of a 2× KAPA HiFi Uracil+ Master Mix, 5.5 μl H_2_O, 1.5 μl DMSO, 1 μl BSA (20 mg/ml), and 1 μl each of a forward and reverse indexing primer (10 μM). Thermocycling conditions were as follows: 5 min at 98°C, followed by 10–12 cycles of 15 sec at 98°C, 20 sec at 60°C, 20 sec at 72°C, and a final 1 min elongation step at 72°C. The enriched libraries were quantified using a TapeStation 2200 (Agilent Technologies) run in high‐sensitivity mode.

### 2.5 High‐throughput sequencing and initial data processing

Preenrichment and postenrichment libraries were pooled separately in equimolar amounts and sequenced on an Illumina HiSeq 2500 in rapid run mode (2 × 150) at the Yale Center for Genome Analysis in West Haven, CT, generating between 1.5 M and 65 M paired‐end reads per library. Illumina sequencing adapters, low quality stretches and leading and trailing Ns were trimmed from the reads using AdapterRemoval 2.0 (Schubert, Lindgreen, & Orlando, 2016). Paired‐end reads with a minimum overlap of 10 bp were collapsed into single reads. After collapsing, reads shorter than 25 bp and nonoverlapping read pairs were discarded. The resulting analysis‐ready reads were mapped to the human reference genome (hg19) using bwa aln (0.7.5a) (Li & Durbin, 2009). To improve the mapping sensitivity of reads with an excess of 5′ terminal substitutions, caused by DNA damage, bwa seeding was disabled (−l = 1,000). Reads with mapping quality lower than 30 were discarded, and PCR duplicates were identified and removed using Picard‐tools 1.130 (http://picard.sourceforge.net). Finally, reads were realigned around indels using Genome Analysis Toolkit 3.3 (DePristo et al., 2011), and the MD‐tag was recalculated using samtools 1.2 (Li et al., 2009). To overcome the problem of uneven pooling prior to sequencing, we selected a random subset of 5 million (5 M) read pairs from each sample for all downstream analyses, unless otherwise noted. For the five dentin samples that produced fewer than 5 M read pairs, we used the total number of reads available (Supporting Information Tables S2 and S3). Summary statistics (depth of coverage, average read length, and GC content) were estimated on the final 5 M dataset alignments using samtools 1.2 (Li et al., 2009).

### 2.6 mapDamage 2.0 analysis

We used *mapDamage2.0* (Jonsson et al., 2013) to quantify postmortem DNA damage patterns (e.g., deamination rates) for each DNA library and to rescale base quality scores of Ts and As based on their probability of resulting from molecular damage.

### 2.7 Contamination estimates

We used *contamMix* 1.05 (Fu et al., 2013) to estimate the level of modern human DNA contamination. In brief, a consensus sequence was built using reads with mapping quality ≥30, base quality ≥20, and a minimum per‐site depth of coverage of three. Additionally, sites with consensus concordance lower than 70% were set to N. Next, mtDNA reads were extracted from the original alignments and re‐mapped to the newly created mtDNA consensus sequence as described above. Then, a second majority count consensus sequence was created from the resulting alignments. Finally, the resulting consensus sequence was aligned to a reference panel containing 311 human mitochondrial DNAs (Green et al., 2008) using *mafft* (Katoh, Misawa, Kuma, & Miyata, 2002) with default parameters and the contamination was estimated using *contamMix*.

### 2.8 Chromosomal sex determination

To determine chromosomal sex, we used the method described by Skoglund et al. (2013), which calculates the fraction of sex chromosome reads that align to the Y chromosome. The analysis was restricted to reads with a mapping quality ≥30.

### 2.9 mtDNA haplogroup assignment

To determine mtDNA haplogroups, mtDNA variant sites were called using samtools 1.2 and bcftools 1.9 (Li et al., 2009), allowing for recalculation of the extended BAQ, and excluding bases with quality lower than 20 and reads with mapping quality lower than 30. Genotypes were compared with the revised Cambridge Reference Sequence (Andrews et al., 1999), and variants with depth of coverage lower than 3 and “allelic balance” lower than 70% were discarded. *HaploGrep2.0* (Weissensteiner et al., 2016) was run on the list of filtered variants to assign the most likely haplogroup for each sample. Because of the low number of mtDNA reads for several individuals, we assessed the accuracy of haplogroup assignment at different depths of coverage by carrying out an in silico downsampling experiment on the full datasets generated for individuals H10 and S40. For each sample, 15,000, 10,000, 5,000, 1,000, 500, 100, and 50 mtDNA reads were randomly subsampled from the total reads, and each downsampled dataset was used to perform haplogroup assignment as described above (Supporting Information Table S4).

### 2.10 Genome‐wide clustering analysis (ADMIXTURE)

We used the model‐based clustering algorithm ADMIXTURE (Alexander, Novembre, & Lange, 2009) to explore the continental level ancestry components of the ancient samples, using the Human Genome Diversity Panel (HGDP) as a reference. Given the low depth of human genome coverage in the ancient samples (Supporting Information Tables S2 and S3), we did not rely on called genotypes, but rather used a genotype likelihood‐based approach to estimate the ancestry proportions, based on the allele frequencies inferred for the modern genotype panel. To do so, we first ran ADMIXTURE (Alexander et al., 2009) on the reference panel assuming three ancestral populations (*K* = 3). In order to prevent suboptimal solutions, we performed 100 replicates with a different seed value and kept the replicate with the highest likelihood. Then for each of the ancient samples, we estimated the three possible genotype‐likelihoods (GL) at the sites included in the reference panel, using the GATK model implemented in ANGSD v.0.614 (Korneliussen et al., 2014). Bases with a quality score lower than 20 and reads with mapping quality lower than 30 were excluded from the analysis. Finally, we estimated the ancestry proportions in the ancient samples using an expectation maximization method (Skotte, Korneliussen, & Albrechtsen, 2013), as implemented in *fastNGSadmix* (Jørsboe, Hanghøj, & Albrechtsen, 2017). For dentin sample S40, we explored three different datasets: data derived from shotgun sequencing, from WGE and the combination of both. Additionally, for sample H10 we explored the effect of sequencing depth in the accuracy of the admixture proportion estimates. To do so, we randomly sampled 5,000,000, 1,000,000, 500,000, 100,000, 50,000, 10,000, and 5,000 reads from the mapped and filtered reads, and obtained 10 independent replicates for each number of reads. For each dataset, we estimated admixture proportions assuming three populations as previously described, and compared the obtained values with the expected proportions estimated on all available reads (Supporting Information Figure S2).

### 2.11 Microbial profiling

Analysis‐ready reads from the full dataset were aligned locally (‐‐no‐unal ‐‐local) to the subset of the Silva SSU 111 reference dataset (Quast et al., 2013) using *Bowtie2* (Langmead & Salzberg, 2012) using default parameters. Reads mapping to the 16S rRNA gene were then assigned to OTUs following the closed‐reference OTU pipeline in QIIME v.1.8 (Caporaso et al., 2010) using 97% similarity threshold with “uclust” (Edgar, 2010) and 500 max accepts, 500 max rejects and Greengenes 13.8 as the reference database (DeSantis et al., 2006). Resulting OTU tables were rarefied to 1,697 (lowest number of reads assigned to OTUs for dental calculus samples), and were summarized at the taxonomic level of phylum.

## 3 RESULTS

### 3.1 Ancient DNA recovery

**Figure 2:**
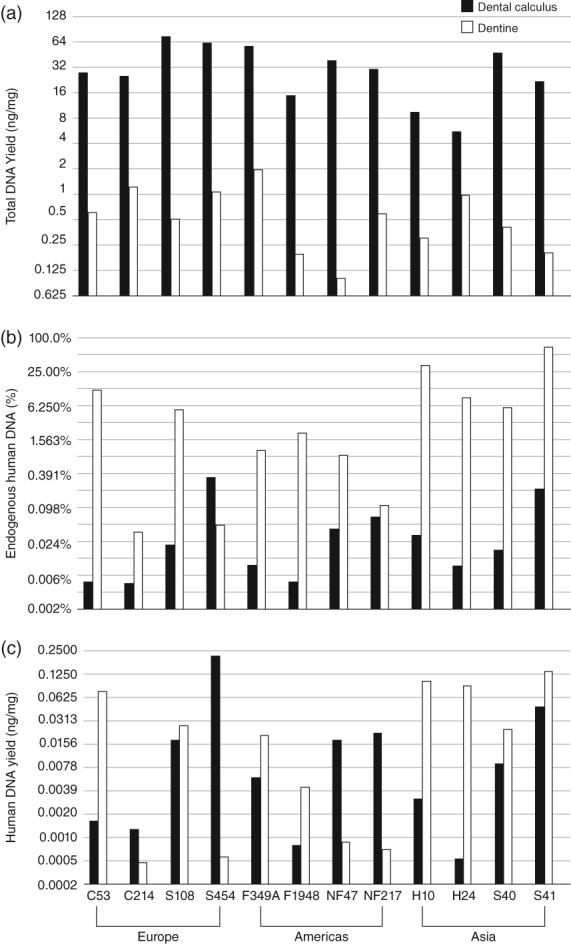
Ancient DNA recovery and human endogenous content from archaeological dental calculus. Bar charts summarizing: (**a**) Total DNA yield (ng/mg) in dental calculus (filled bars) and dentin samples (hollow bars) on a log 2 scale. Dental calculus samples show a higher total DNA yield compared with dentin samples in all cases. (**b**) Endogenous human DNA content (%) in dental calculus (filled bars) and dentin samples (hollow bars) on a log 2 scale. Overall human endogenous content is higher for dentin samples. (**c**) Estimated absolute human DNA yields (ng/mg) on a log 2 scale. Total human DNA per mg is estimated to be higher in dental calculus in 4 out of 12 sample pairs, but both substrates exhibit high variation. Archaeological sites are ordered from left to right by continent (Europe, Americas, Asia). Data are provided in Supporting Information Table S2

The total amount of DNA extracted from dental calculus (5.4–72.1 ng/mg) significantly exceeded that recovered from dentin (0.1–1.9 ng/mg) in all cases (Wilcoxon signed‐rank test, *p* < .001; Figure 2a; Supporting Information Table S2), a finding consistent with previous studies (Mann et al., 2018; Ozga et al., 2016; Warinner et al., 2014). The DNA in dental calculus was primarily of microbial origin, but human reads were also present, and the relative proportion of human DNA was significantly lower in dental calculus (0.005–0.35%) than dentin (0.04–66.95%; Wilcoxon signed‐rank test, *p* < .01; Figure 2b; Supporting Information Table S2). When normalized by sample mass input, however, the estimated absolute quantity of human DNA in dental calculus (0.5–210 pg/mg) and dentin (0.5–130 pg/mg) was similar (Wilcoxon signed‐rank test, *p* > .3), albeit highly variable, with 4 of 12 sample pairs containing more human DNA in dental calculus, and eight sample pairs containing more human DNA in dentin (Figure 2c). As expected, we observed that the human DNA in dental calculus samples from older contexts (i.e., Camino del Molino, Spain) or warmer climates (i.e., Anse à la Gourde, Guadeloupe) were generally less well‐preserved (as indicated by lower human DNA contents, shorter average fragment lengths, and higher deamination rates) than the DNA recovered from younger samples or those from colder climates (e.g., Nepal or the Netherlands). However, contrary to our expectations we did not observe the same pattern for the dentin samples (Supporting Information Table S2).

### 3.2 Whole human genome enrichment

**Figure 3:**
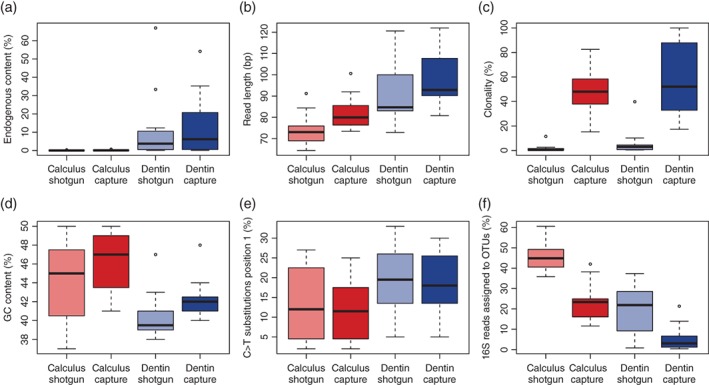
Box plots showing the impact of whole genome capture on sequencing results in the 5 M dataset. (**a**) The proportion of human DNA (%) increases in the capture dataset by approximately four‐fold for both dental calculus and dentin. Three dentin samples (S41, F349A, and C53) did not show any enrichment or declined in human DNA content following capture. (**b**) Fragment lengths (bp) are longer in the capture dataset for both dental calculus and dentin. (**c**) Clonality significantly increases as a result of capture for both substrates. (**d**) GC content (%) is significantly higher for dental calculus samples and is higher than the average GC content of the human genome. After capture, GC content increases in both dentin and dental calculus libraries. (**e**) Cytosine deamination (C > T) at 5′ position one of the reads is slightly lower in calculus than dentin and does not change as a result of whole human genome enrichment. (**f**) The proportion of 16S rDNA reads assigned to OTUs (%) decreases after capture for both dental calculus and dentin. Data are available in Supporting Information Tables S2 and S3

Whole genome enrichment resulted in uneven enrichments in the human DNA content of both dentin and dental calculus libraries and for three of the dentin libraries (S108, F349A, and S41) capture did not lead to any significant enrichment at all (see Table 1). Generally, we observed higher enrichment factors for the dental calculus libraries than the dentin libraries, but due to the low starting amount of human DNA in the dental calculus libraries the absolute gains were relatively low (Figure 3a). Enrichment of mitochondrial DNA (up to 140‐fold) was higher than nuclear DNA (up to four‐fold) (Supporting Information Tables S2 and S3), but still relatively low when compared with previous studies specifically targeting the mitochondrial genome (Ozga et al., 2016).

**Table 1:** Endogenous human DNA content in percent (%) before and after whole genome capture for 12 paired dentin and dental calculus libraries

Sample	Dentin			Calculus		
	Precapture	Postcapture	Enrichment	Precapture	Postcapture	Enrichment
C53	12.3	16.4	1.3	0.01	0.02	4.2
C214	0.0	0.1	2.5	0.01	0.02	3.7
S108	5.4	4.7	0.9	0.02	0.09	3.5
S454	0.1	0.1	2.4	0.35	0.54	1.6
F349A	1.0	0.7	0.7	0.01	0.03	3.1
F1948	2.1	7.6	3.6	0.01	0.02	4.4
NF47	0.9	2.3	2.7	0.05	0.13	2.8
NF217	0.1	0.4	3.1	0.07	0.28	4.0
H10	33.4	35.3	1.1	0.03	0.11	3.2
H24	8.7	25.1	2.9	0.01	0.03	2.6
S40	6.0	15.3	2.5	0.02	0.04	2.1
S41	66.9	54.2	0.8	0.22	0.70	3.2

As observed elsewhere (Mann et al., 2018), average human DNA fragment lengths in the precapture libraries were found to be significantly shorter (Wilcoxon signed‐rank test, *p* < .01) in dental calculus (73 bp) compared with dentin (85 bp) (Figure 3b), and dental calculus samples exhibited a significant increase in average fragment length after capture (dental calculus 80 bp; dentin 93 bp) (Wilcoxon signed‐rank test, *p* < .01). However, no correlation was found between fragment length in precapture libraries and capture success (enrichment), nor was there any correlation between fragment length in precapture libraries and endogenous human DNA content (Wilcoxon signed‐rank test, *p* > .01).

Clonality markedly increased with capture in both dentin and dental calculus (Wilcoxon signed‐rank test, *p* < .001 for dental calculus; *p* < .01 for dentin) (Figure 3c), and the GC content of human reads was significantly higher in postcapture than precapture libraries (Wilcoxon signed‐rank test, *p* < .01; Figure 3d). The GC content of human reads in dental calculus (median 45% in precapture and 47% postcapture libraries) was also significantly higher than in dentin (median 40% in precapture and 42% postcapture libraries; Wilcoxon signed‐rank test, *p* < .05 for both precapture and postcapture). To ensure that the elevated GC content of human reads in dental calculus was not a consequence of mismapping of bacterial reads (which have a higher median GC content) to the human genome, we performed a BLASTn search of 10,000 randomly sampled dental calculus human reads against the NCBI nt database, and confirmed that most (97–98%) uniquely mapped to human (Supporting Information Figure S1).

Terminal damage rates were significantly lower in dental calculus than in dentin (Wilcoxon signed‐rank test, *p* = .01), and WGE did not significantly influence damage rates (Wilcoxon signed‐rank test, *p* > .1) (Figure 3e).

### 3.3 Contamination estimates

Due to the low sequencing depth per sample, we were only able to estimate mitochondrial contamination rates for half of the dentin samples and none of the calculus samples. In the precapture dataset, contamination estimates were generally low and ranged from 0.6 to 4.1% (Supporting Information Table S2). After capture, we observed higher contamination rates, ranging from 1 to 15.7% (Supporting Information Table S3).

### 3.4 Sex identification

Previous studies (Skoglund et al., 2013) suggest that a minimum of 3,000 reads mapping to the sex chromosomes are required to accurately identify the chromosomal sex of ancient remains from high‐throughput sequencing data. For the calculus libraries, we recovered less than 300 sex chromosome reads per sample prior to capture. This increased slightly after capture, to a maximum of 892 reads for S41, but was still not sufficient to obtain reliable estimates (Table 2). For the dentin libraries, we recovered significantly more sex chromosome reads, averaging around 13,000 reads per sample prior to capture, and 17,000 reads after capture (Table 2). This was sufficient to obtain reliable sex estimates for 8 of the 12 individuals (Table 2, of which, 4 were identified as female and 4 as male.

**Table 2:** Reads mapping to sex chromosomes in precapture and postcapture libraries from 5 M dataset

Sample	Precapture				Postcapture			
	X + Y^a^	Ry^b^	SE	Sex^c^	X + Y^a^	Ry^b^	SE	Sex^c^
*Calculus*								
C53	8	n.d.	n.d.	n.d.	54	n.d.	n.d.	n.d.
C214	11	n.d.	n.d.	n.d.	25	0.040	0.039	Female^d^
S108	19	0.158	0.084	Male^d^	73	0.055	0.027	Male^d^
S454	279	0.118	0.019	Male^d^	447	0.119	0.015	Male^d^
F349A	6	n.d.	n.d.	n.d.	34	0.118	0.055	Male^d^
F1948	20	n.d.	n.d.	n.d.	45	n.d.	n.d.	n.d.
NF47	64	n.d.	n.d.	n.d.	274	0.007	0.005	Female^d^
NF217	65	0.154	0.045	Male^d^	327	0.107	0.017	Male^d^
H10	44	0.091	0.043	Male^d^	146	0.151	0.030	Male^d^
H24	14	0.071	0.069	Male^d^	33	0.182	0.067	Male^d^
S40	34	n.d.	n.d.	n.d.	66	0.015	0.015	Female^d^
S41	207	0.087	0.020	Male^d^	892	0.148	0.012	Male^d^
*Dentine*								
C53	25,168	0.004	0.001	Female	40,096	0.013	0.001	Female
C214	93	0.022	0.015	Female^d^	151	0.026	0.013	Female^d^
S108	5,141	0.096	0.004	Male	4,765	0.117	0.005	Male
S454	35	0.086	0.047	Male^d^	33	0.151	0.062	Male^d^
F349A	1,142	0.096	0.009	Male^d^	955	0.157	0.012	Male^d^
F1948	4,404	0.005	0.001	Female	16,675	0.009	0.001	Female
NF47	1,493	0.009	0.002	Female^d^	4,144	0.015	0.002	Female
NF217	121	0.099	0.027	Male^d^	454	0.121	0.015	Male^d^
H10	34,928	0.096	0.002	Male	36,034	0.129	0.002	Male
H24	10,014	0.095	0.003	Male	10,668	0.134	0.003	Male
S40	10,719	0.004	0.001	Female	31,031	0.012	0.001	Female
S41	66,225	0.093	0.001	Male	64,243	0.116	0.001	Male

a Total number of reads mapping to the sex chromosomes after removing PCR duplicates and reads with mapping quality <30.

b Ry observed fraction of Y chromosome alignments compared with the total number of alignments to the X and Y chromosome (Skoglund et al., 2013).

c Typical Ry for males is an Ry over 0.09. Ry values under 0.02 are considered female.

d Sex predicted by Ry value, but insufficient X + Y reads are available for confident assignment. A minimum of 3,000 R + Y reads are recommended for sex assignment (Skoglund et al., 2013).

### 3.5 Mitochondrial haplogroup determination

Similar to obtaining sex estimates from high‐throughput sequencing data, a minimum number of reads mapping to the mitochondrial genome are needed to confidently assign a mitochondrial haplogroup. To determine how many mitochondrial reads are needed, we serially downsampled the total mitochondrial reads of two well‐preserved dentin samples, H10 and S40, from approximately 15,000 to 50 mitochondrial reads and observed how this affected haplogroup assignment and scoring using the program HaploGrep (Weissensteiner et al., 2016). We found that at least approximately 500 mitochondrial reads are needed to obtain haplogroup assignments with a HaploGrep score greater than 0.5, and at least 1,000 mitochondrial reads are recommended for more confident assignments (Supporting Information Table S4). Depending on aDNA fragment length, this corresponds to an average depth of coverage of approximately 1–4×. However, we note that other factors, such as the number of sequencing errors, deamination rates (postmortem damage), and contamination will also affect the accuracy of the haplogroup assignment.

As expected, we recovered significantly more mitochondrial reads from dentin than from dental calculus (Wilcoxon signed‐rank test, *p* < .001 for both precapture and postcapture libraries; Supporting Information Table S2). Whole genome capture led to substantial enrichments of mitochondrial reads (6‐ to 139‐fold for dental calculus and 2‐ to 99‐fold for dentin) (Table 1). However, even with capture we still did not recover enough mitochondrial reads from the dental calculus libraries to confidently determine mitochondrial haplogroups, and only one sample, NF217, allowed a low confidence assignment to haplogroup A2 (HaploGrep score 0.5). For dentin, five of the 12 samples yielded sufficient mitochondrial reads without capture to determine the haplogroup with a HaploGrep score greater than 0.6 (Supporting Information Table S5). After capture, we were also able to obtain haplogroup assignments for five out of the 12 samples. The assignments were consistent before and after capture, and the identified haplogroups are found among contemporary populations in the sampled regions today (Supporting Information Table S5).

### 3.6 Genome‐wide ancestry estimation

**Figure 4:**
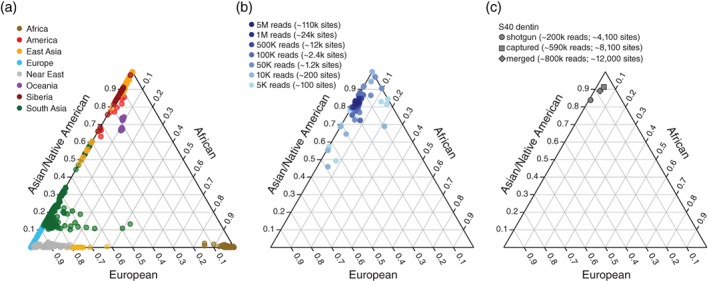
Genome‐wide ancestry estimates. (**a**) Overview of reference populations from the HGDP panel used in the ADMIXTURE analysis. (**b**) Results of a downsampling experiment for one sample in our dataset (H10 dentin) illustrating how genome‐wide ancestry estimates become increasingly unreliable as the amount of data decreases (dark blue to light blue). (**c**) Genome‐wide ancestry estimates based on ADMIXTURE results (shown at *K* = 3) for sample S40 dentin estimated using ADMIXTURE. Plotted are the results for the data derived from shotgun sequencing (circle), after whole genome enrichment (square), and the combination of both (diamond)

Ancestry estimation programs like ADMIXTURE (Alexander et al., 2009) rely on reference panels to provide genome‐wide ancestry estimates for ancient samples. Among other things, the accuracy of these estimates depends on the intersection between the data that were generated from the ancient samples and the reference panel used. Simply speaking, the larger the overlap is, the more accurate the estimates are. Using a series of randomly downsampled datasets generated from the total human reads of dentin sample H10 and the Human Genome Diversity Panel (HGDP) as a global reference, we found that at least 100,000 total mapping nuclear reads (corresponding to ~2,500 overlapping sites) are needed in order to obtain consistent admixture proportions at *K* = 3 with low standard errors using the clustering algorithm ADMIXTURE (Figure 4a,b; Supporting Information Figure S2) (Alexander et al., 2009). Below 100,000 reads, the estimated admixture proportions became increasingly variable with large standard errors and, therefore, unreliable.

In accordance with the mtDNA results, we recovered significantly more nuclear reads from dentin than calculus (Wilcoxon signed‐rank test, *p* < .01 for precapture and postcapture libraries). However, at a sequencing depth of 5 M paired‐end reads, only 6 out of 12 (precapture) and 7 out of 12 (postcapture) dentin samples and none of the calculus samples yielded more than 100,000 autosomal reads. Whole genome capture generally led to modest enrichments in autosomal reads for both dental calculus (five‐fold) and dentin (four‐fold) and, therefore, also led to greater overlap between the ancient samples and the reference panel. In cases where the capture worked, we recovered roughly twice as many sites after capture (Supporting Information Tables S2 and S3), given the same sequencing effort, which also improved the accuracy of the ADMIXTURE‐based ancestry estimates. This can be most clearly seen in the case of S40 dentin where the results from ADMIXTURE analysis performed on the precapture dataset erroneously assigned a higher proportion of European ancestry to an individual of Asian ancestry (Figure 4c).

### 3.7 Microbial profile

The effect of WGE on off‐target microbial DNA sequences was evaluated by comparing the taxonomic assignment of 16S rRNA gene sequences (16S rDNA) before and after human enrichment on the total sequencing dataset. Contrary to expectations, the proportion of 16S rDNA sequences did not decrease in the captured libraries, as might be expected for this off‐target and relatively high GC content gene (Supporting Information Tables S2 and S3). However, taxonomic analysis of these 16S rDNA reads using the QIIME pipeline (Caporaso et al., 2010) revealed that the proportion of 16S rDNA reads assignable to OTUs using a closed reference 97% identity clustering approach did decrease significantly from 45.5 to 23.1% for dental calculus and from 19.6 to 5.3% for dentin (Wilcoxon signed‐rank test, *p* < .00003 for dental calculus, *p* < .004 for dentin; Figure 3f; Supporting Information Table S2). These results are broadly consistent with analyses performed using the MetaBIT pipeline (Louvel et al., 2016), where the median postcapture OTU assignment rate fell by half for dental calculus (from 44.9 to 23.4%) and by more than four‐fifths for dentin (from 21.9 to 3.1%) (Supporting Information Tables S2 and S3). Although higher rates of OTU assignment for dental calculus compared with dentin in both precapture and postcapture libraries are not unexpected because the host‐associated taxa present in dental calculus are better represented in reference databases than the environmental taxa present in dentin, the reason for the overall drop in OTU assignment rates observed after capture is unclear.

**Figure 5:**
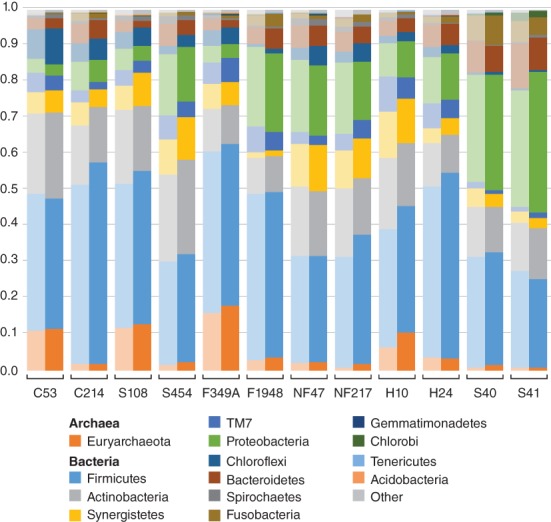
Frequency of microbial phyla inferred from dental calculus samples before and after whole genome capture. The microbial profiles prior to (light) and after (dark) human genome capture enrichment were similar between precapture and postcapture libraries, indicating that off‐target microbial sequences from postcapture libraries may be suitable for use in ancient oral microbiome studies

As a consequence of the very low numbers of 16S rDNA reads recovered from the dentin samples, further taxonomic analysis was restricted to the dental calculus samples only. Precapture and postcapture dental calculus datasets were rarefied to a depth of 1,697 and assigned taxonomy using the QIIME pipeline and the Greengenes database (Caporaso et al., 2010). Despite significant differences in OTU assignment rates before and after capture, overall phylum‐level taxonomic proportions were similar between precapture and postcapture libraries (Figure 5). This suggests that off‐target microbial sequences obtained through WGE of dental calculus may be suitable for phylum‐level microbial community structure analysis of the ancient human oral microbiome.

## 4 DISCUSSION

The principal aim of this study was to evaluate whether dental calculus could serve as a viable alternative source of human DNA for whole human genome reconstruction and to explore the efficacy of WGE on dental calculus. To do so, we characterized the human DNA content in a diverse set of archaeological dental calculus and dentin samples, before and after WGE. In agreement with previous studies (Mann et al., 2018; Ozga et al., 2016; Warinner et al., 2014), we observed that the total extracted DNA yield of dental calculus far exceeds that of dentin—up to 375‐times higher as in the case of NF47. Additionally, we found that although the proportion of human DNA in the samples is generally lower in dental calculus than dentin, the absolute amount of human DNA in both substrates is comparable (cf., Mann et al., 2018).

Whole genome capture resulted in up four‐fold enrichment of the human endogenous content in both dental calculus and dentin libraries, with the exception of three dentin samples that either showed no enrichment (S108) or, in fact, were depleted in human DNA content (F349A, S41) following capture. These enrichments are comparable to those observed in previous studies using a whole genome capture approach (Carpenter et al., 2013; Schroeder et al., 2015; Ávila‐Arcos et al., 2015; Schroeder et al., 2018), but significantly lower than those reported for mitochondrial genome capture (Ozga et al., 2016) or the targeted capture of specific SNPs (Mathieson et al., 2015). This suggests that current WGE methods are not as effective as other forms of targeted enrichment, which might be at least partially explained by the size and complexity of the target, the low copy number of nuclear DNA (when compared with mtDNA), the presence of repetitive sections in the genome, and the presence of homologous regions in nonhuman/bacterial taxa.

Previous studies have reported that an initial endogenous content of 1–25% resulted in higher enrichment factors (Ávila‐Arcos et al., 2011; Cruz‐Dávalos et al., 2016); however, we found no correlation between initial endogenous content and enrichment. The overall low levels of enrichment observed in this study for both dentin and dental calculus samples suggest that current WGE techniques are not efficient at enriching the human DNA in either sample material.

To further investigate the effects of in‐solution capture on ancient DNA libraries, we compared variables such as contamination, average fragment length, clonality, GC content, and deamination rate before and after capture (Supporting Information Tables S2 and S3). As observed previously, we find slightly higher contamination estimates for postcapture libraries (Supporting Information Tables S2 and S3) (Ávila‐Arcos et al., 2015), and we found that postcapture average fragment lengths were longer (Supporting Information Tables S2 and S3), which is consistent with the known bias toward longer fragments when using a capture approach (Cruz‐Dávalos et al., 2016; Ozga et al., 2016). While we found no correlation between average DNA fragment length and initial endogenous human DNA content or capture performance (enrichment rate), we agree with previous studies that fragment length might be a limiting factor when applying WGE on extremely degraded ancient DNA samples (Cruz‐Dávalos et al., 2016; Enk et al., 2014). In addition, the significantly shorter (Wilcoxon signed‐rank test, *p* < .01) average human DNA fragment lengths in the precapture dental calculus libraries could be the result of the active incorporation of human DNA in dental calculus through host inflammatory responses and, in particular, the release of neutrophil extracellular traps (NETosis) (Mann et al., 2018).

After capture, we find that clonality increases approximately 50‐fold (Supporting Information Tables S2 and S3). This is likely due to the high number of PCR cycles used for samples with relatively low initial endogenous DNA contents, which has been previously shown to result in increased clonality (Ávila‐Arcos et al., 2015). However, a high number of PCR cycles can be necessary to reach the required amount of input DNA for hybridization capture.

Interestingly, we find that prior to enrichment the median GC content of human DNA in the calculus libraries (45%) is significantly higher than in the dentin libraries (39.5%), as well as higher than the average GC content of the human genome (40.9%). Previous studies have shown that the GC content of DNA retrieved from dentin typically reflects that of the reference genome (Cruz‐Dávalos et al., 2016). However, a recent study (Mann et al., 2018) observed an inverse relationship between DNA fragment length and GC content in ancient DNA derived from microbial taxa. The systematic loss of AT‐rich fragments in taxa with low‐ and medium‐GC genomes may be partially explained by the susceptibility of short fragments with low GC content to loss through denaturation (Mann et al., 2018). The high GC content of human DNA in dental calculus might be related to factors specific to the manner in which human DNA is incorporated into dental calculus. However, it remains unclear whether these patterns are produced through the sequencing preparation or a naturally occurring taphonomic process (Mann et al., 2018). Furthermore, we note that capture slightly increased the overall GC content of the ancient DNA libraries.

Regarding postmortem damage, we did not observe any changes in the frequency of typical damage patterns following enrichment (cf., Carpenter et al., 2013). We did, however, detect significant differences in terminal damage rates between dental calculus and dentin, whereby human DNA in dental calculus appears to be less damaged than in dentin (cf., Mann et al., 2018). It is possible, therefore, that the human DNA trapped in dental calculus is somehow more protected from various degradation processes (e.g., hydrolytic damage) than human DNA in dentin. Overall, despite some differences that may be intrinsic to biological differences between dental calculus and dentin, we find that in‐solution WGE affects dental calculus in a similar way as dentin or bone.

With respect to sex and ancestry estimates, we find that WGE marginally improved the reliability of these assignments by enabling the generation of more data for the same sequencing effort. As such, we recovered approximately twice as many sex chromosome reads on average with WGE than without. This was sufficient to reliably determine the biological sex of 8 of the 12 individuals, 4 of whom were identified as female, and 4 as male. While we were unable to recover sufficient X and Y chromosome reads from the calculus samples to obtain confident sex estimates, we note that sex chromosome reads were present and that given the appropriate sample size and sequencing effort, high‐throughput sequencing of archaeological dental calculus samples could be used for sexing ancient human remains. With respect to genome‐wide ancestry estimates, we note that WGE increased the overlap between the ancient samples and the modern reference panel and, therefore, also improved the accuracy of the ADMIXTURE‐based ancestry estimates. We also recovered significantly more mitochondrial reads after capture, resulting in more reliable mtDNA haplogroup estimates.

Finally, in regard to the sample microbial profile, we found that although the proportion of 16S rDNA reads assigned to OTUs significantly decreased after capture, no major differences were observed in microbiome profiles at the phylum level, indicating that off‐target reads in libraries enriched for the human genome may still be useful for investigating the ancient oral microbiome.

Overall, we note that our WGE experiment was notably less effective than other forms of capture targeting the mitochondrial genome (Maricic et al., 2010; Ozga et al., 2016) or specific SNPs (e.g., Mathieson et al., 2015). We believe that this might at least partially be due to the small starting amount of target DNA present in many of the samples. In addition, the efficiency of WGE might be limited by the size and complexity of the human genome, the low copy number of nuclear DNA (when compared with mtDNA), the presence of repetitive sections in the genome, and the presence of homologous regions in nonhuman, including bacterial taxa. Regardless of the precise cause, further optimization is clearly needed before WGE can be more widely applied. Two possible modifications for in‐solution based WGE include (1) increasing the amount of starting DNA, and (2) optimizing hybridization temperatures and incubation times. For example, Paijmans et al. (2016) found the best results with hybridization temperatures of 65°C for degraded samples, while Cruz‐Dávalos et al. (2016) found that longer incubation times (40 hr instead of 24) at lower temperatures (50°C) led to higher enrichment rates. A third option would be to perform two or more consecutive rounds of capture (Li et al. 2013; Templeton et al., 2013) to increase enrichment rates.

## 5 CONCLUSION

Whole human genome capture performed on a set of 24 paired human dental calculus and dentin samples resulted in up to four‐fold enrichments of the human endogenous content. These kinds of enrichment rates are orders of magnitude lower than those achieved with other kinds of capture targeting the mitochondrial genome (Maricic et al., 2010; Ozga et al., 2016) or specific SNPs (Mathieson et al., 2015). We conclude that while archaeological dental calculus does contain ancient human DNA, current WGE techniques are not effective at retrieving it, and further optimizations are needed before WGE can be more widely applied. The low relative proportion of human DNA in dental calculus clearly poses challenges for retrieving host genome information using both shotgun and capture enrichment approaches. However, in the absence of other resources or when sampling of other tissues is restricted, dental calculus can serve as a viable source of human ancient DNA. The ability to recover both human DNA and microbial DNA from the same archaeological substrate opens new avenues of research for studying the relationships between the genetic information of the host and microbiome composition, function and evolution. However, to fully realize the potential of dental calculus for human genome‐wide analyses, optimization of DNA enrichment techniques is necessary.

## AUTHOR CONTRIBUTIONS

H.S. and C.W. designed the research. K.Z. and A.O. performed the experiments. J.R.M., K.Z., A.E.M., K.S., C.H., B.B., C.W., and H.S. analyzed the data. C.L.H., C.M.L., M.H., D.S.G., B.F., G.R.M., A.S., and M.A. provided materials and resources. K.Z., J.R.M., C.W., and H.S. wrote the manuscript, with input from all the other authors.

## Supporting information

Appendix S1: Supplementary Tables

Appendix S1: Supplementary Figures
