# Supplementary material for: The efficacy of whole human genome capture on ancient dental calculus and dentin
Source: Am J Phys Anthropol. 2018 Dec 26;168(3):496–509. doi: 10.1002/ajpa.23763 (PMC6519167; doi:10.1002/ajpa.23763)
Supplement: Supplementary file 2 — Appendix S1: Supplementary Figures [file AJPA-168-496-s002.docx]

**Supplementary Figures**

**Supplementary Figure 1.** Results of BLASTn search of putative human reads against the NCBI nt database for F349A dental calculus and dentin. BLAST was used to test whether human assigned reads were human-specific or could be high GC content microbial mis-assignments that falsely map to the human genome. The results of this analysis support the identification of our human-assigned reads as human in origin.

**
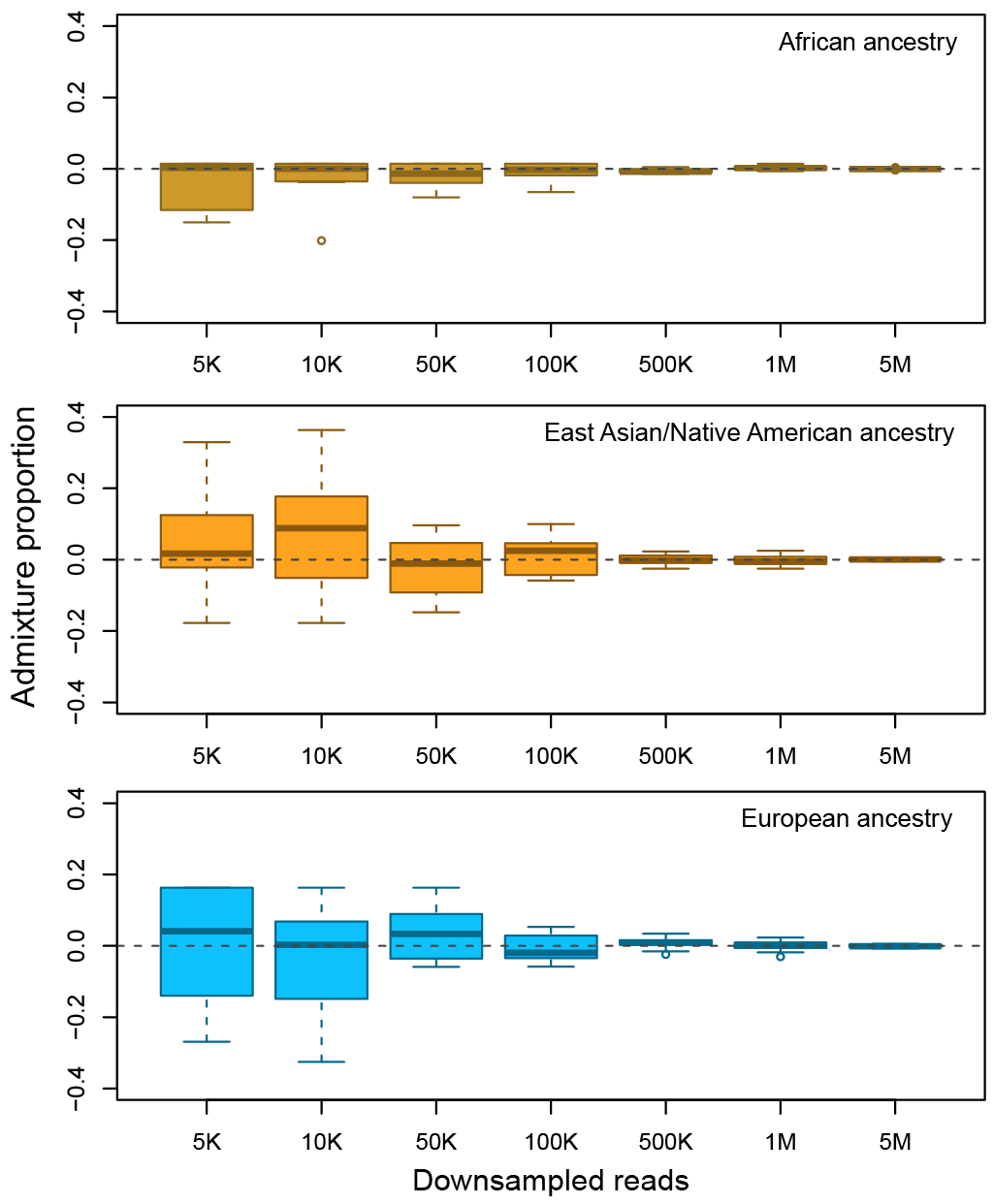
**

**Supplementary Figure 2.** Box plots showing the deviation from the expected admixture proportions in each of 10 replicates and for different number of subsampled reads. For dentin sample H10, we randomly sampled 5,000 (5K), 10,000 (10K), 50,000 (50K), 100,000, (100K), 500,000 (500K), 1,000,000 (1M) and 5,000,000 (5M) reads and obtained ten independent replicates of each dataset. Box plot displays the difference between the expected (estimated using all available data) and the observed (estimated in the corresponding replicate) admixture proportions for each of the 3 ancestries. A grey dotted line marks *y=0*.
